# Application of TaqMan Real-Time PCR for Detecting ‘*Candidatus* Arsenophonus Phytopathogenicus’ Infection in Sugar Beet

**DOI:** 10.3390/pathogens10111466

**Published:** 2021-11-12

**Authors:** Christina Zübert, Michael Kube

**Affiliations:** Department of Integrative Infection Biology Crops-Livestock, University of Hohenheim, 70599 Stuttgart, Germany; christina.zuebert@uni-hohenheim.de

**Keywords:** TaqMan qPCR, ‘*Candidatus* Arsenophonus phytopathogenicus’, SBR, sugar beet

## Abstract

The γ-proteobacterium ‘*Candidatus* Arsenophonus phytopathogenicus’ is assigned as the major pathogen of “Syndrome des basses richesses”, a sugar beet disease characterised by a reduction in the sugar content of taproots and biomass yield. Despite the economic impact of this bacteriosis, diagnostics for this important pathogen currently rely on end-point PCR detection. Herein, we introduce a TaqMan qPCR for diagnostics of the agent targeting genes encoding a heat shock protein of the Hsp20 family and mannose-6-phosphate isomerase. Quantitation with synthetic oligonucleotides as standard showed that the developed TaqMan qPCR assays enable the detection of up to 100 target copies. A comparison between the TaqMan qPCR and end-point PCR for ‘*Ca.* A. phytopathogenicus’ detection was carried out on 78 sugar beet samples from different locations in southern Germany. The newly developed assays enable the fast, reliable and sensitive detection of ‘*Ca*. A. phytopathogenicus’ in sugar beet.

## 1. Introduction

Germany is one of the world’s largest sugar beet (*Beta vulgaris*) producers, but in 2008, a new and devastating disease, named “Syndrome des basses richesses” (SBR), was recognised in the country [1]. SBR has been well-known in neighbouring French Burgundy since the 1990s and results in taproots characterised by a lowered sugar content of up to 5% and reduced biomass, resulting in high economic losses [2]. It is now widespread in south-western parts of Germany, where it threatens profitability for sugar beet growers and associated industry sectors. Two vector-transmitted and phloem-limited bacteria are associated with this disease, namely the γ-proteobacterium ‘*Candidatus* Arsenophonus phytopathogenicus’ [3,4,5] and ‘*Candidatus* Phytoplasma solani’ from the Mollicutes class [2,6]. ‘*Ca.* A. phytopathogenicus’ has been identified as the main agent for SBR [7,8], and is transmitted by the planthopper *Pentastiridius leporinus* (*Cixiidae*) [4,6]. Phytoplasma infection of sugar beet by ‘*Ca.* P. solani’ has also been reported for Germany [9], but the epidemiology remains unclear.

Additionally, infected sugar beet plants show yellowing and necrotic old leaves, while small, narrow and chlorotic leaves are produced by new shoots. The taproot is characterised by a brown-coloured phloem [3]. Besides the visual inspection of sugar beets, several molecular assays have been developed for detecting ‘*Ca.* A. phytopathogenicus’, including the application of the primer pair Fra4/Fra5, targeting the 16S rRNA gene [9], originally designed for the closely-related ‘*Candidatus* Phlomobacter fragariae’ that causes the marginal chlorosis of strawberries [10]. The usage of the primer pair led to the discovery of ‘*Ca.* A. phytopathogenicus’ [3]. Fra5 can also be applied in combination with the universal bacterial primers fD1/rP1 in PCR, in order to generate longer amplification products [4,7]. Furthermore, the primers Alb1/Oliv1 have been designed to amplify ‘*Ca.* A. phytopathogenicus’ 16S-ITS region, resulting in five DNA fragments with a characteristic pattern of four bands [11]. Additional primer sets for end-point PCR have been designed to characterise the *spoT*-*spoU*-*recG* gene locus [12]. Unfortunately, no qPCR assay has been published to date. The purpose of this study was to evaluate two TaqMan qPCR assays for detecting ‘*Ca.* A. phytopathogenicus’ for beet samples in different German SBR regions. 

## 2. Results

Two TaqMan assays were developed to target the mannose-6-phosphat isomerase and heat shock protein 20 genes for detecting and quantifying ‘*Ca.* A. phytopathogenicus’. The evaluation of 78 taproot samples provided evidence on the reliability of not only the newly developed TaqMan qPCR assays (this study), but also the application of an established Fra4/Fra5 endpoint-PCR assay [3]. 

### 2.1. Primers and Probes for qPCR

Primers targeting *manA* and the *hsp20* gene of ‘*Ca.* A. phytopathogenicus’ produced amplification products of 116 bp and 90 bp, respectively, while the designed *nad5* sugar beet plant template control assay for *Beta vulgaris* generated a 96 bp amplification product (Table 1). Partial gene sequences show the highest sequence similarities to the *Arsenophonus* endosymbionts of *Aphis craccivora* (CP038155.1, CP038156.1) with 98% for *manA* and 95% for *hsp20* identity. 

### 2.2. Detection Limit for the ‘Ca. A. Phytopathogenicus’-Specific TaqMan Assay 

**Figure 1:**
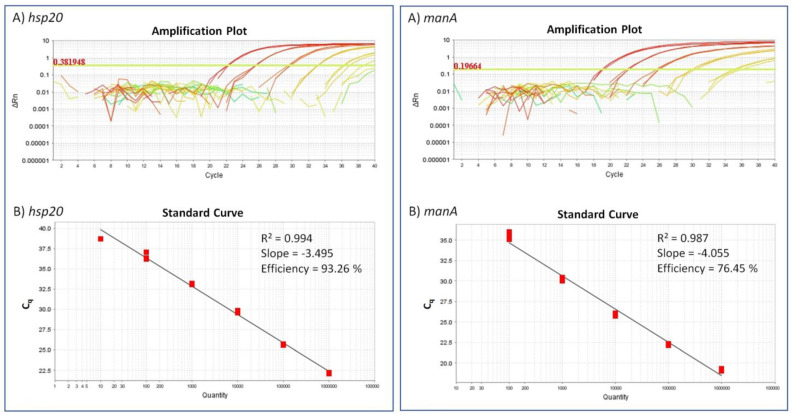
Amplification plot (**A**) and standard curve (**B**) for the *hsp20* and *manA*-TaqMan qPCR assays. A dilution series (10^6^ to 10^0^) of gBlocks was measured in triplicate. (**A**) Amplification plots for the gBlock dilutions and NTCs. (**B**) Standard curve obtained by plotting the threshold numbers of PCR cycles (triplicates) against the dilution (log scale).

The application of diluted gBlocks resulted in standard curves with an R^2^ value of 0.994 and 0.987 for *hsp20* and *manA*, respectively. The slopes of the linear fit showed values of −3.495 and −4.055, thereby resulting in an efficiency of 93.26% for *hsp20*- and 76.45% for *manA*-TaqMan assay (Figure 1).

For the *hsp20* TaqMan assay, mean quantification cycle (C_q_) values, previously known as the threshold cycle (C_t_), ranged between 22.2 for the lowest dilution (1.0 × 10^6^) and 38.7 for the dilution of 10 copies (1.0 × 10^1^), but only one reaction of the three replicated with 10 copies was identified as positive. Reactions with one copy (1.0 × 10^0^) showed no amplification. The variation in C_q_ increased at 100 copies to a standard deviation (C_q_ SD) of 0.37 for *hsp20*. A concentration between 1.0 × 10^6^ and 1.0 × 10^3^ copies/reaction led to consistent amplification (standard deviation ≤ 0.12 cycles), while serial dilution steps from 1.0 × 10^6^ to 1.0 × 10^1^ copies differed by a mean C_q_ value of 3.3. 

The *manA*-TaqMan assay resulted in mean C_q_ values (triplicate) ranging from 19.2 (1.0 × 10^6^) to 35.5 (1.0 × 10^2^). Copy numbers 10 and 1 were not identified. The mean C_q_ values of serial dilutions differed by an average of 4.07. As for the *hsp20* assay, at 100 copies there was no consistent amplification (C_q_ SD of 0.41). A concentration between 1.0 × 10^6^ and 1.0 × 10^3^ copies/reaction led to consistent amplification (standard deviation ≤ 0.19 cycles). For both assays, samples with C_q_ values < 40 can be considered positive. 

In summary, both assays reliably detected 10^2^ target copies. The *hsp20* assay showed better efficiency in the evaluation, but within the standard curve measuring, the *manA*-assay showed higher C_q_ values in comparison to the *hsp20* assay. 

### 2.3. TaqMan qPCR versus End-Point PCR

Both TaqMan qPCR assays were consistent in identifying ‘*Ca.* A. phytopathogenicus’ in 59 out of 78 taproot samples (19 negative samples). The C_q_ values of the positive samples were compared to the those obtained from the standard *manA* and *hsp20* TaqMan assay curves. Most of the positive samples (44 out of 59 for *hsp20* and 45 out of 59 for *manA*) had mean C_q_ values < 30 (Supplementary Material Table S1), indicating copies of min. 1.0 × 10^3^ or higher (Table 2). Despite differences in the efficiency of the two assays for detecting the agent, screened sugar beet samples showed comparable C_q_ values for both assays. 

The plant control, targeting *nad5* of *B. vulgaris*, showed strong amplification, with C_q_ values ranging from 16.0 to 21.3 for the examined samples. Unfortunately, this assay was not suitable for combination with *manA* or *hsp20* assay primers for multiplexing. Depending on primer concentrations, we either produced non-detectable results for SBR or it resulted in high Cq values close to the maximum number of cycles for both the internal control (*nad5*) and the SBR targets genes (*hsp20* and *manA*) (data not shown). Therefore, application of the *nadA* assay is suggested, in order to confirm the presence of a usable template in the assays.

Identification of ‘*Ca.* A. phytopathogenicus’ taproot samples by the TaqMan assays was confirmed by end-point PCR using the primer pair Fra4/Fra5 [10], resulting in the expected 550 bp PCR products (Supplementary Material Figure S1). 

### 2.4. Sampling Sites and Verification of the Amplification Products

Twelve sampling sites in five federal states were selected in Germany (Table 3). Ten sampling sites contained positive taproot samples. For two of the 12 sampling sites, all taproot samples were negative (Bondorf and Wendershausen). 

Sequences of 13 samples from ten positive sampling sites were amplified in end-point PCR (Fra4/Fra5) and sequenced thereafter. A multiple sequence alignment of samples sequenced with Fra4/Fra5 showed 100% identical sequences over 486 bp, which matched the expected part of the 16S ribosomal gene in the sequence analysis.

Seven positive samples from six sampling sites were amplified and sequenced with TaqMan assay primers (*hsp20* and *manA*); additionally, 12 samples from all sampling sites were chosen for verifying amplified sequences with the internal primer *nad5*. Multiple sequence alignment analysis of the fragments showed 100% identity between all sequences (from different sampling sites) for each primer pair. Sequence analyses confirmed the desired amplification targets in all cases.

## 3. Discussion

For more than a decade, SBR has spread across southern Germany. It completely covers some sugar beet plots, while others are visually asymptomatic and—as exemplified herein—remain uninfected in October 2020 (Bondorf and Wendershausen). The fast, sensitive and reliable detection of ‘*Ca.* A. phytopathogenicus’ is one of the major requirements for gaining a better understanding of the epidemiology. Pressure on sugar beet cultivation in Germany is not limited to ‘*Ca.* A. phytopathogenicus’, however. Several viruses caused by Beet yellows virus (BYV), beet mild yellowing virus (BMYV), beet chlorosis virus (BChV) and beet mosaic virus (BtMV) cause virus yellows (VY) disease in sugar beet, resulting in high economic losses [13]. Other bacterioses of sugar beet have been recently analysed, including the Rubbery Taproot Disease caused by ‘*Candidatus* Phytoplasma solani’ in the Pannonian Plain or a related strain from Germany [9]. The latter was discovered during a routine screening of sugar beet whilst applying a widely used phytoplasma 16S-rDNA TaqMan assay with a detection limit originally described at 10^6^ [14]. In a recent study, this assay was re-evaluated in comparison to a specific assay for detecting ‘*Candidatus* Phytoplasma ulmi’ in elm species [15]. Results not only strongly supported the reliability of the 16S-rDNA TaqMan assay, but it also shows that the assay reaches a detection limit of ten copies and a C_q_ value of 37.2. This result was obtained in parallel to a specific assay for detecting ‘*Candidatus* Phytoplasma ulmi’, reaching a minimum of ten template copies with a C_q_ value of 35.1 and using a plasmid DNA standard supplemented by elm DNA. In these studies, the calculation of the standard curve was also based on a 10-fold dilution of the quantified target. An approach for a more precise estimation for the limit of detection would be to narrow down the target concentration of standards while increasing the number of replicates, as suggested by Forootan et al., 2017 [16]. One may speculate about other factors influencing the detection limit, but in this regard, consideration should be given to the impact of the applied template, probe labelling, enzyme mixes, microtiter plates, the real-time cycler, etc. In any case, the impact of the phytoplasma 16S-rDNA TaqMan assay in phytoplasma diagnostics will not be touched [14]. Here, the assays failed to reach a detection limit of ten copies in the triplicate. It cannot be estimated if this is related to the usage of gBlocks as standards. These synthetic controls are popular due to their defined sequences, the option for quick ordering and the possibility of easy exchange between research teams [17]. Previously, this approach has been used for the quarantine pest pathogen *Xylella fastidiosa*, enabling a detection of ten copies and achieving a C_q_ value of 35 [18]. However, in contrast, the authors applied pure *X. fastidiosa* DNA and a gBlock standard not supplemented with plant DNA. The impact of supplementation by plant templates on qPCR efficiency remains unclear, but similar C_q_ values of 36.5 (*hsp20*) and 35.5 (*manA*) have been achieved in comparison. 

As shown above, many studies, including this one, reach detection limits at high C_q_ values. In accordance with other studies [18], samples are considered to contain the pathogen if they show a C_q_ value of <40 and an amplification curve with exponential growth. 

The TaqMan qPCR assays presented here enable the reliable and early detection of infection as well as quantification. This prerequisite offers the fast screening of sugar beet genotypes exhibiting weak infection or tolerance despite high colonisation; however, it also enables the identification of so-far unknown vectors and reservoir plants that need to be considered in phytosanitary management. These possibilities should not be underestimated with respect to already announced SBR-tolerant sugar beet varieties.

## 4. Materials and Methods

### 4.1. Plant Material

A total of 78 sugar beet plants were obtained from ten fields in the federal states of Baden-Wuerttemberg (BW), Rhineland-Palatinate (RP), Bavaria (BY), Saxony (SN) and Hesse (HE) in Germany (Table 3). 

### 4.2. DNA Templates

Taproot tissue (2 g) was homogenised in plastic extraction bags (Bioreba, Grenzach, Switzerland), and nucleic acid extraction was performed using the cetyltrimethylammonium bromide extraction procedure [19,20]. The purified pellet was dissolved in 75 μL of double-distilled water and stored at −20 °C.

The absolute concentration of the DNA samples was measured using a Qubit® fluorometer with a dsDNA assay BR Assay Kit (Invitrogen, Carlsbad, Germany) according to the manufacturer’s instructions. All samples were diluted prior to amplification to an average DNA amount of 3.4 ng/µL and transferred in 96-well plates. 

### 4.3. Gene Target Selection and Primer Design for the TaqMan qPCR Assay 

Primers and associated TaqMan probes were designed in Primer 3 release 4.1.0 (https://primer3.ut.ee accessed on 10 February 2020) [21], using default parameters but setting the product size range from 90–130 bp. The ‘*Ca.* A. phytopathogenicus’ target gene sequences, mannose 6-phosphate isomerase (*manA*) and heat shock protein 20 (*hsp20*), were obtained via a metagenomic shotgun applying DNA from an infected sugar beet sample, since no genome sequence of the pathogen is available in public databases. The target sequences belong to a small subset of the metagenomic data assigned to the pathogen and fulfil the criteria for the selection of suitable oligonucleotide sets. Gene sequences were deposited in GenBank under the accession numbers OK335756 and OK335757. Target sequences identical to other entries in GenBank have been searched by BLASTN [22]. The internal oligos (TaqMan probes) were dual-labelled with 6′-carboxyfluorescein (5’ end) and a Black Hole Quencher-1 (3′-end) (6′FAM/BHQ-1). Primers and probes for plant control were designed on a *Beta vulgaris* NADH-ubiquinone oxidoreductase chain 5 encoding gene (*nad5*) sequence from GenBank (NC_002511.2, BevupMp052), and the probe was labelled with hexachlorofluorescein at the 5′ end and a Black Hole Quencher-1 at the 3′-end (HEX/BHQ-1). All oligonucleotides (Table 1) were obtained from Metabion (Planegg/Steinkirchen, Germany).

### 4.4. TaqMan Assay Conditions

Each TaqMan qPCR reaction comprised 2.08 µL double distilled water, 10 µL of Fast Probe qPCR Master Mix (Roboklon, Berlin, Germany), 0.45 µM of each primer and 0.2 µM probe, 0.2 µL Uracil-N-Glycosylase (UNG) and 7.5 µL of diluted DNA template (approximately 26 ng).

The cycling parameters comprised uracil-N-glycosylase digested at 37 °C for 2 min, an initial denaturation step at 95 °C for 3 min, followed by 40 cycles of a two-step protocol of 95 °C for 15 s and 60 °C for 30 s. Real-time PCR was conducted on a StepOnePlus Real-Time PCR System (Applied Biosystems, Darmstadt, Germany). Samples including four negative template controls were measured as triplets. Data evaluation was done via StepOne Software v2.3 (Applied Biosystems, Darmstadt, Germany). 

### 4.5. Quantification and Evaluation of TaqMan qPCRs, Using gBlocks

The detection limit was evaluated by constructing a standard curve, using synthetic oligonucleotides (gBlocks). The double-stranded gBlock fragments (Integrated DNA Technologies, Leuven, Belgium) are 125 bp long (Table 4).

The lyophilised gBlock fragments were dissolved in 0.1 × TE-Buffer. The stock solution provided over 1.8 × 10^12^ copies and was diluted to receive 1.0 × 10^6^ copies/µL, followed by a serial dilution 1:10 to 1.0 × 10^0^ for each gBlock. Seven dilution levels (1.0 × 10^6^ copies to 1 copy in 2 µL) and a non-template control (NTC, double distilled water), each with three replicates, were measured. The results were also verified by repeatedly measuring with DNA (29.3 ng) from a non-infected taproot added to dilution steps 1.0 × 10^6^, 1.0 × 10^4^, 1.0 × 10^2^, 1.0 × 10^1^/PCR reaction to obtain similar conditions to those for unknown samples. 

The standard curves were created by plotting the Ct-values of dilution series against the relative concentration on a semi-logarithmic scale via StepOne Software v2.3 (Applied Biosystems, Darmstadt, Germany). The R square and slope values were calculated based on standard curves, while the reaction efficiency of the assay was evaluated with the formula E_X_ = 10^(−1/slope)^. The baseline was always set automatically, and the threshold for the *hsp20* TaqMan assay was set manually at 0.381948. The standard curve was used to assess sensitivity and to estimate roughly the bacteria titer in the samples.

### 4.6. End-Point PCR and Gel Electrophoresis

The results of the TaqMan qPCRs were compared to those of the same samples measured in end-point PCR, targeting part of the 16S rDNA sequence [10]. Sugar beet samples are considered to be SBR-positive in qPCR if the C_q_ value is <40, while positive detection in end-point PCR is relying on visualisation of amplification products by agarose gel electrophoresis. PCRs were performed in a reaction volume of 25 µL, including 12.5 µL OptiTaq Master Mix (Roboklon, Berlin, Germany), 0.08 µL of each primer Fra4/Fra5 (100 µM), 4.84 µL double-distilled water and 7.5 µL DNA. Cycling conditions consisted of an initial denaturation step at 95 °C for 90 s, 35 cycles of 92 °C, 52 °C and 72 °C each of 1 min and a final extension at 72 °C for 1 min. The obtained PCR products (550 bp) were separated in agarose gel (1%), stained with GelRed (Genaxxon bioscience, Ulm, Germany) and visualised with a UV transilluminator.

### 4.7. Verification of Amplification Products 

Amplification products for the TaqMan assay and conventional end-point PCR [10] were confirmed by Sanger sequencing (Macrogen Europe B.V., Amsterdam, The Netherlands) the amplification products of the SBR_manAF/SBR_manA, SBR_hsp20F/SBR_hsp20R, BV_nad5F/BV_nad5R and Fra4/Fra5 primer set (Table 1). 

## Figures and Tables

**Table 1:** TaqMan primers and probes designed for detecting ‘*Ca.* A. phytopathogenicus’ and *B. vulgaris* as plant controls for the gene targets heat shock protein 20 (*hsp20*), mannose 6-phosphat isomerase (*manA*) and NADH-ubiquinone oxidoreductase chain 5 (*nad5*).

Species	Amplicon Size	Oligonucleotide	Length	Sequence (5′–3′)	Labelling
*Ca.* P. phytopathogenicus	90	SBR_hsp20_F	21	CACTTTTGCCGCTGATAGTCA	
		SBR_hsp20_R	20	TGGAACTCACAGTAGCGGTT	
		SBR_hsp20_P	23	AACTCCTGTTGTTTATAACCAGG	6-FAM/BHQ-1
*Ca.* P. phytopathogenicus	116	SBR_manA_F	20	CAACCAGGTGAAGCGATGTT	
		SBR_manA_R	20	TTGTTAGTTAATCCCGCGCG	
		SBR_manA_P	20	TCTCTATGCCAGAACTCCGC	6-FAM/BHQ-1
*B. vulgaris*	96	BV_nad5_F	20	TGAATGACGAGTCGGACCAA	
		BV_nad5_R	20	TCGGAGAGCACTGAATTCGA	
		BV_nad5_P	20	TACCCTTGCGTGCAATGATG	HEX/BHQ-1

**Table 2:** Mean C_q_ values (±SD) for the gBlock standards after qPCR. Detection limit in triplicate is 100 copies/reaction (outlined in red).

Copy Number	C_q_ Values of the *hsp20*-Target ^a^	C_q_ Values of the *manA*-Target ^a^
1 × 10^6^	22.2 (±0.07)	19.2 (±0.12)
1 × 10^5^	25.7 (±0.05)	22.2 (±0.10)
1 × 10^4^	29.7 (±0.12)	25.9 (±0.15)
1 × 10^3^	33.1 (±0.04)	30.1 (±0.19)
1 × 10^2^	36.5 (±0.37)	35.5 (±0.41)
1 × 10^1^	not detected (38.7 ^b^)	not detected
1 × 10^0^	not detected	not detected

^a^ Mean of three technical replicates; ^b^ C_q_ value of one technical replicate, the other two technical replicates are not detected.

**Table 3:** Sampling sites with coordinates in DD format (decimal degrees) and qPCR results.

Sampling Site	Variety	No of Samples	*manA*-qPCR *	*hsp20*-qPCR *	Coordinates
Gundelsheim (BW)	Eucalyptus	7	29 (28–32)	29 (26–31)	49.264944, 9.160611
Bondorf (BW)	Alcedo	3	not detected	not detected	48.528556, 8.821111
Wendershausen (HE)	LUNELLA KWS	16	not detected	not detected	51.323417, 9.882556
Massenbach (BW)	BTS 440	7	32 (29–38)	30 (28–33)	49.177556, 9.063472
Gemmingen (BW)	Racoon	5	31 (27–34)	30 (26–32)	49.159167, 9.003556
Fürfeld (BW)	Raison	7	28 (27–30)	28 (27–29)	49.212389, 9.052028
Bickenbach (HE)	BTS 8750 N	5	30 (29–32)	30 (28–30)	49.760708, 8.602995
Ochsenfurt, Gollhofen (BY)	LUNELLA KWS	6	28 (27–29)	28 (27–29)	49.577722, 10.184778
Heddesheim (BW)	BTS7300	6	27 (26–29)	28 (25–29)	49.509024, 8.583888
Ochsenfurt, Rodheim (BY)	BTS 440	6	30 (28–33)	30 (28–33)	49.587222, 10.150194
Deutschhof (Südpfalz) (RP)	N/A	6	28 (25–30)	28 (26–29)	49.086707, 8.020107
Welsau (SN)	ADVENA KWS	4	30 (28–32)	29 (28–32)	51.577121, 12.950829

* Mean C_q_ values (each sample measured in triplicate) and range of all C_q_ values (min–max).

**Table 4:** Sequences of double-stranded gBlock fragments for *hsp20* and *manA* target.

Target	Sequence (5′–3′)
*hsp20*	5′-GTTTCACTTTTGCCGCTGATAGTCAGCTTATTATTCAATACTGATATATCTAACTCCTGTTGTTTATAACCAGGAACCGCTACTGTGAGTTCCAGATTTTCTTTATCTTTTTGGTATAAATTGTA-3′
*manA*	5′-GAATTACAACCAGGTGAAGCGATGTTTCTCTATGCCAGAACTCCGCATGCTTATATTGAAGGTGTTGGTTTAGAAGTAATGGCCAATTCTGACAATGTACTGCGCGCGGGATTAACTAACAAACA-3′.

## Data Availability

Gene sequences were deposited in GenBank under the accession numbers OK335756 and OK335757.

## Supplementary Materials

The following are available online at https://www.mdpi.com/article/10.3390/pathogens10111466/s1, Figure S1: Gel images of end-point PCR using Fra4/Fra5 primers, Table S1: C_q_ values of TaqMan qPCR for the gene targets heat shock protein 20 (*hsp20*), mannose 6-phosphat isomerase (*manA*) and NADH-ubiquinone oxidoreductase chain 5 (*nad5*).
