# Supplementary material for: Application of TaqMan Real-Time PCR for Detecting ‘Candidatus Arsenophonus Phytopathogenicus’ Infection in Sugar Beet
Source: Pathogens. 2021 Nov 12;10(11):1466. doi: 10.3390/pathogens10111466 (PMC8625364; doi:10.3390/pathogens10111466)
Supplement: Supplementary file 1 [file pathogens-10-01466-s001.zip › pathogens-1429055-supplementary/Zuebert_Kube_Figure_S1.pdf]

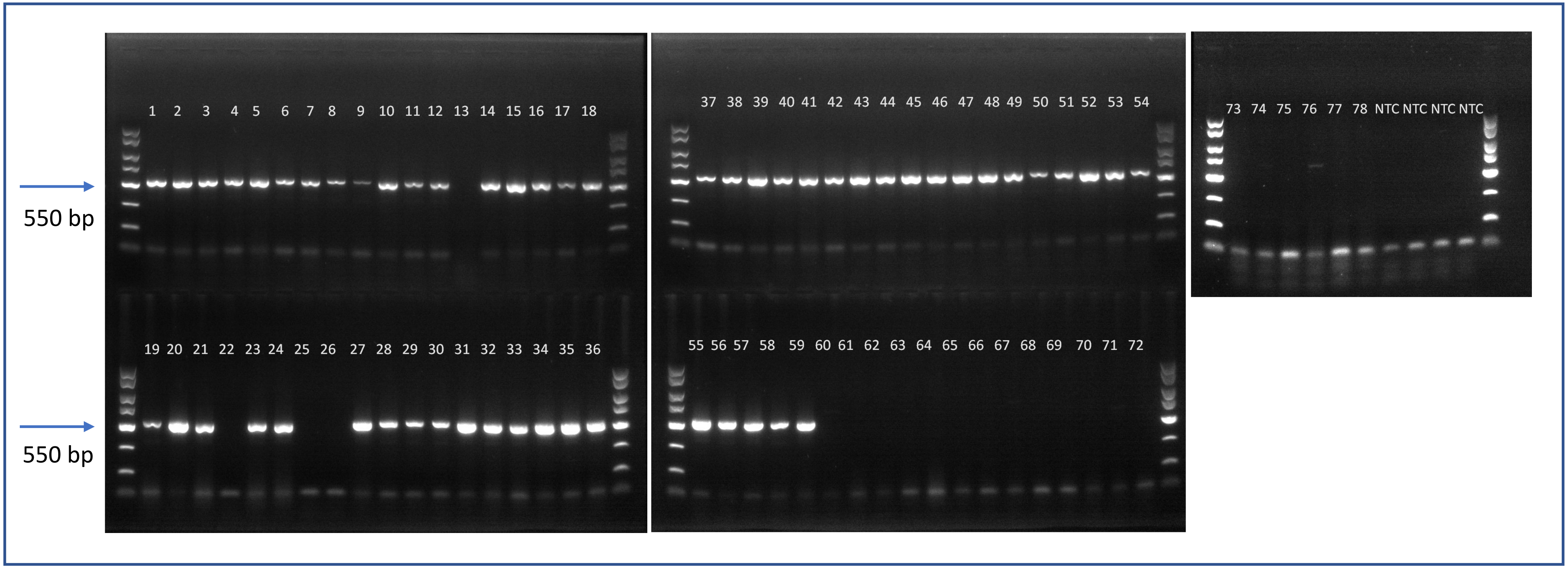

Figure S1: Gel images of end-point PCR using Fra4/Fra5 primers. Four samples failed in the first round of end-point PCR, due to a technical issue with the robot system. Repetition of these PCRs provided the expected products without any change in conditions.
