# Supplementary material for: Application of TaqMan Real-Time PCR for Detecting ‘Candidatus Arsenophonus Phytopathogenicus’ Infection in Sugar Beet
Source: Pathogens. 2021 Nov 12;10(11):1466. doi: 10.3390/pathogens10111466 (PMC8625364; doi:10.3390/pathogens10111466)
Supplement: Supplementary file 1 [file pathogens-10-01466-s001.zip › pathogens-1429055-supplementary/Zuebert_Kube_Table_S1_041021.pdf]

Table S1: Ct values of TaqMan qPCR for the gene targets heat shock protein 20 (*hsp20*), mannose 6-phosphat isomerase (*manA*) and NADH-ubiquinone oxidoreductase chain 5 (*nad5*).

		manA				*hsp20*				*nad5*
**Sample No.**	**Sampling Site**	**Run 1**	**Run 2**	**Run 3**	**Mean ct**	**Run 1**	**Run 2**	**Run 3**	**Mean ct**	**Run 1**
Sample 1	Gundelsheim	30,1	29,0	29,4	29,5	28,8	28,5	25,9 *	27,7	17,5
Sample 2	Gundelsheim	28,7	28,7	29,4	28,9	28,3	28,5	28,1	28,3	17,5
Sample 3	Gundelsheim	28,4	28,4	29,0	28,6	28,7	28,8	28,5	28,7	18,5
Sample 4	Gundelsheim	28,2	28,3	28,8	28,4	28,6	28,7	28,6	28,6	17,7
Sample 5	Gundelsheim	28,7	28,3	28,0	28,3	28,9	28,6	28,3	28,6	17,8
Sample 6	Gundelsheim	29,0	29,4	29,5	29,3	29,4	29,0	28,8	29,1	19,0
Sample 7	Gundelsheim	32,0	31,4	31,5	31,6	31,0	30,7	29,0	30,2	17,6
Sample 8	Massenbach	31,9	32,3	32,2	32,1	31,2	30,8	30,8	30,9	19,1
Sample 9	Massenbach	35,7	35,4	38,1	36,4	33,1	33,0	32,0	32,7	19,7
Sample 10	Massenbach	29,9	29,2	29,4	29,5	28,8	28,7	28,2	28,6	19,4
Sample 11	Massenbach	34,7	34,2	36,3	35,1	32,0	32,1	31,6	31,9	19,2
Sample 12	Massenbach	30,2	30,3	28,4	29,6	30,2	27,5	29,3	29,0	18,6
Sample 13	Massenbach	33,3	32,2	33,5	33,0	31,4	31,5	30,9	31,2	19,4
Sample 14	Massenbach	30,1	30,3	30,3	30,2	28,9	28,6	28,5	28,7	18,4
Sample 15	Gemmingen	27,2	27,4	27,5	27,3	27,0	26,9	25,7	26,5	17,4
Sample 16	Gemmingen	29,7	29,9	30,7	30,1	29,2	29,2	28,9	29,1	19,4
Sample 17	Gemmingen	33,1	33,3	34,2	33,5	31,8	31,8	31,1	31,5	17,9
Sample 18	Gemmingen	29,6	29,5	30,2	29,8	29,3	29,3	29,0	29,2	19,3
Sample 19	Gemmingen	32,1	31,4	34,0	32,5	31,0	30,9	30,6	30,8	18,0
Sample 20	Fürfeld	26,5	26,5	26,6	26,6	27,1	27,0	26,7	26,9	19,7
Sample 21	Fürfeld	28,8	29,0	29,1	29,0	28,6	28,6	28,3	28,5	17,1
Sample 22	Fürfeld	27,6	27,4	27,6	27,5	26,5	26,9	23,8	25,7	16,2
Sample 23	Fürfeld	28,5	28,5	28,6	28,5	29,1	28,6	28,4	28,7	17,9
Sample 24	Fürfeld	28,4	28,1	28,3	28,2	28,0	28,1	27,7	27,9	17,5
Sample 25	Fürfeld	29,0	29,6	29,5	29,4	28,6	28,5	28,6	28,6	18,8
Sample 26	Bickenbach	30,3	31,1	32,4	31,2	30,3	30,3	30,1	30,2	18,3
Sample 27	Bickenbach	29,7	29,4	29,2	29,4	28,7	28,9	28,4	28,7	18,0
Sample 28	Bickenbach	31,4	31,2	31,9	31,5	30,3	30,3	30,1	30,2	20,4
Sample 29	Bickenbach	29,0	28,8	28,7	28,8	29,7	29,7	29,1	29,5	20,3
Sample 30	Bickenbach	29,4	29,9	29,3	29,5	30,3	30,4	30,1	30,3	20,3
Sample 31	Ochsenfurt, Gollhofen	26,7	27,1	26,8	26,9	27,2	27,1	26,6	27,0	18,7
Sample 32	Ochsenfurt, Gollhofen	28,8	29,0	29,1	29,0	29,1	28,8	28,7	28,9	19,3
Sample 33	Ochsenfurt, Gollhofen	29,4	28,7	29,2	29,1	28,8	28,7	28,5	28,7	18,4
Sample 34	Ochsenfurt, Gollhofen	27,6	27,3	27,3	27,4	27,6	27,7	27,2	27,5	19,4
Sample 35	Ochsenfurt, Gollhofen	27,4	27,3	26,8	27,2	27,7	27,6	27,4	27,6	19,1
Sample 36	Ochsenfurt, Gollhofen	28,4	28,6	28,3	28,4	28,6	28,5	28,1	28,4	18,5
Sample 37	Deutschhof (Südpfalz)	28,9	29,9	30,1	29,6	29,4	29,0	29,1	29,2	19,0
Sample 38	Deutschhof (Südpfalz)	28,2	29,4	28,9	28,8	28,0	28,3	28,1	28,1	19,1
Sample 39	Deutschhof (Südpfalz)	25,4	25,2	25,2	25,3	25,8	25,8	25,5	25,7	19,2
Sample 40	Deutschhof (Südpfalz)	28,5	27,8	28,4	28,2	28,5	28,2	28,2	28,3	18,8
Sample 41	Deutschhof (Südpfalz)	23,8	26,5	26,6	25,6	26,8	27,0	26,7	26,8	18,4
Sample 42	Deutschhof (Südpfalz)	28,0	28,3	28,4	28,2	28,5	28,2	28,1	28,3	19,6
Sample 43	Heddesheim	25,5	25,8	25,7	25,7	25,6	25,7	24,8	25,4	20,6
Sample 44	Heddesheim	28,1	27,9	28,7	28,2	28,8	28,6	28,4	28,6	19,7
Sample 45	Heddesheim	27,0	27,2	27,4	27,2	27,5	27,6	27,0	27,4	20,5
Sample 46	Heddesheim	27,8	28,1	27,8	27,9	27,6	27,5	27,2	27,4	19,7
Sample 47	Heddesheim	27,8	27,5	27,2	27,5	28,3	28,3	27,7	28,1	19,7
Sample 48	Heddesheim	28,4	27,8	27,0	27,7	28,2	27,7	27,7	27,9	20,4
Sample 49	Ochsenfurt, Rodheim	29,6	30,1	29,9	29,9	30,1	30,0	30,0	30,0	18,8
Sample 50	Ochsenfurt, Rodheim	32,4	32,7	33,1	32,7	32,5	32,3	32,6	32,4	19,4
Sample 51	Ochsenfurt, Rodheim	29,9	29,9	29,8	29,9	30,2	30,5	30,1	30,3	19,6
Sample 52	Ochsenfurt, Rodheim	27,8	27,9	27,8	27,8	28,5	28,1	28,0	28,2	20,1
Sample 53	Ochsenfurt, Rodheim	28,8	28,7	28,3	28,6	29,1	29,2	29,3	29,2	19,4
Sample 54	Ochsenfurt, Rodheim	31,0	31,1	30,9	31,0	30,9	30,9	30,9	30,9	20,5
Sample 55	Welsau	28,5	28,2	27,8	28,2	28,0	27,6	27,6	27,7	21,2
Sample 56	Welsau	30,0	30,0	29,4	29,8	29,6	29,3	28,8	29,2	21,3
Sample 57	Welsau	29,2	28,9	28,6	28,9	28,9	28,6	28,5	28,7	21,1
Sample 58	Welsau	32,3	32,4	31,6	32,1	31,7	31,6	31,4	31,6	19,4
Sample 59	Fürfeld	28,2	28,5	27,9	28,2	28,4	28,2	28,1	28,2	18,5
Sample 60	Wendershausen	40,0	40,0	40,0	40,0	40,0	40,0	40,0	40,0	20,1
Sample 61	Wendershausen	40,0	40,0	40,0	40,0	40,0	40,0	40,0	40,0	18,0
Sample 62	Bondorf	40,0	40,0	40,0	40,0	40,0	40,0	40,0	40,0	18,0
Sample 63	Bondorf	40,0	40,0	40,0	40,0	40,0	40,0	40,0	40,0	18,0
Sample 64	Bondorf	40,0	40,0	40,0	40,0	40,0	40,0	40,0	40,0	16,1
Sample 65	Wendershausen	40,0	40,0	40,0	40,0	40,0	40,0	40,0	40,0	18,7
Sample 66	Wendershausen	40,0	40,0	40,0	40,0	40,0	40,0	40,0	40,0	18,7
Sample 67	Wendershausen	40,0	40,0	40,0	40,0	40,0	40,0	40,0	40,0	18,3
Sample 68	Wendershausen	40,0	40,0	40,0	40,0	40,0	40,0	40,0	40,0	18,2
Sample 69	Wendershausen	40,0	40,0	40,0	40,0	40,0	40,0	40,0	40,0	18,0
Sample 70	Wendershausen	40,0	40,0	40,0	40,0	40,0	40,0	40,0	40,0	18,7
Sample 71	Wendershausen	40,0	40,0	40,0	40,0	40,0	40,0	40,0	40,0	18,9
Sample 72	Wendershausen	40,0	40,0	40,0	40,0	40,0	40,0	40,0	40,0	18,5
Sample 73	Wendershausen	40,0	40,0	40,0	40,0	40,0	40,0	40,0	40,0	17,4
Sample 74	Wendershausen	40,0	40,0	40,0	40,0	40,0	40,0	40,0	40,0	16,2
Sample 75	Wendershausen	40,0	40,0	40,0	40,0	40,0	40,0	40,0	40,0	20,7
Sample 76	Wendershausen	40,0	40,0	40,0	40,0	40,0	40,0	40,0	40,0	18,5
Sample 77	Wendershausen	40,0	40,0	40,0	40,0	40,0	40,0	40,0	40,0	17,5
Sample 78	Wendershausen	40,0	40,0	40,0	40,0	40,0	40,0	40,0	40,0	18,2
NTC 1		40,0	40,0	40,0	40,0	40,0	40,0	40,0	40,0	40,0
NTC 2		40,0	40,0	40,0	40,0	40,0	40,0	40,0	40,0	37,9
NTC 3		40,0	40,0	40,0	40,0	40,0	40,0	40,0	40,0	40,0
NTC 4		40,0	40,0	40,0	40,0	40,0	40,0	40,0	40,0	40,0
